# Delivery of Mental Health Services in Bhutan: Challenges and Way Forward

**DOI:** 10.1002/puh2.211

**Published:** 2024-06-30

**Authors:** Kelzang Gyeltshen, Bikram Chhetri, Dawa Gyeltshen

**Affiliations:** ^1^ Department of Psychiatry Jigme Dorji Wangchuck National Referral Hospital Thimphu Bhutan; ^2^ Department of Medicine Jigme Dorji Wangchuck National Referral Hospital Thimphu Bhutan

**Keywords:** Bhutan, human resources, mental health services, mental health, stigma

## Abstract

Bhutan is known for its developmental philosophy of Gross National Happiness. Bhutan introduced mental health services in 1997 with the launch of Mental Health Program. Mental health includes psychological, emotional, and social well‐being. The Department of Psychiatry, Jigme Dorji Wangchuck National Referral Hospital in the capital city Thimphu is the only psychiatric unit providing specialized mental health services in Bhutan. The psychiatric unit consists of multidisciplinary team rendering both out‐ and in‐patient mental health services. However, mental health services were gradually integrated in district hospitals, Primary Healthcare Centres and in schools. Although mental health services are an essential component of the healthcare system, the system faces numerous difficulties. Inaccessibility to mental health services due to geographical location and inadequate mental health professionals poses challenges in addressing mental health issues. Mental health issues are perceived differently, and the people with mental health issues often suffer from stigmatization creating barriers in seeking mental health services. Therefore, misconception and stigma associated to mental illness also cause greater challenges in delivering mental health services. Mental health funding has been substantially lower than the World Health Organization recommended mental health budget, amounting to 1% of health budget. Inadequate mental health funding remained a serious concern for the last few decades. Adequate mental health funding is vital in expanding the overall Mental Health Program and improving both the quality and quantity of mental health professionals. Additionally, enhancing community mental health initiatives will reinforce mental health services. Furthermore, there is a need for advancements in evidence and mental health research to support evidence‐based practices and enhance mental health literacy.

## Graphical abstract

Geographical inaccessibility, human resource constraints, stigma, and inadequate funding are some of the challenges in addressing mental health issues in Bhutan. The potential way forward includes increasing human resources, and mental health literacy, decentralizing mental health services, strengthening community mental health research, and increase funding in mental health.

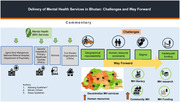

## 1 Introduction

Bhutan is known for its emphasis on Gross National Happiness (GNH). Driven by the developmental philosophy of GNH, the constitution mandates free access to crucial healthcare services, with the government covering the expenses, including those for healthcare services and referrals to a third country [1]. Mental health services in Bhutan became known only in 1997 with the launch of mental health program by the Ministry of Health (MoH) [2]. World Health Organization (WHO) sponsored Burmese psychiatrist, Dr. U. Thuta was the first psychiatrist to serve until 1999 when the first Bhutanese psychiatrist, Dr. Chencho Dorji joined the Jigme Dorji Wangchuck (JDW) National Referral Hospital [2].

Mental health is an integral part of health and well‐being. According to the WHO, mental health is defined as a state of well‐being that enables person to cope with the normal stresses of life, realize his/her abilities, can work, and contribute productively to the community [3]. It includes our emotional, psychological, and social well‐being [3]. Mental health issues are very common in all nations [3, 4]. Around one in eight persons worldwide suffer from a mental illness [4]. In Bhutan, the Annual Health Bulletin 2023 reported a total of 11,038 people with mental and behavioral disorders [5]. Among these, anxiety disorder was reported the highest, with 3441 cases. Mental and behavioral disorders due to alcohol and depression are among the other top cases of mental illnesses. Moreover, alcohol liver disease, attributed to alcohol use disorder, has been one of the leading causes of mortality for more than two decades [5]. Mental illness contributes to poor health outcomes, premature death, human rights violations, and global and national economic loss.

The COVID‐19 pandemic has exacerbated the impact on mental health and well‐being of people in Bhutan with substantial increase in the mental health problems [1, 6]. In the year 2021, the mental health disorders increased to 156 per 10,000 population from 87 per 10,000 population in 2016. Specifically, the prevalence of depression and anxiety showed an unprecedented upward trend during the pandemic. Depression increased from an average rate of 9 per 10,000 individuals between 2011 and 2019 to 16 per 10,000 in 2020 and 32 per 10,000 in 2021. Similarly, anxiety saw a rise from an average rate of 18 per 10,000 between 2011 and 2019 to 29 per 10,000 in 2020 and 55 per 10,000 in 2021 [1]. The rise in the mental health issues was attributed to the unprecedented national lockdowns and uncertainty like growing unemployment, school closure, online schooling pressure, school dropout, and increased domestic violence triggered by COVID‐19 pandemic [1, 6].

According to World Mental Health Report 2022, the needs for mental health are far‐reaching but the responses are inadequate. Other health conditions are often prioritized over mental health, and community mental health (CMH) service is consistently underfunded [4]. On average, countries around the globe dedicate less than 2% of their healthcare budgets to mental health [4]. Bhutan, over the last 5 years, has only dedicated 1% of its healthcare budget to mental health [1]. Around half of world's population lives in countries where there is just one psychiatrist to serve 200,000 or more people [4]. Additionally, there are not many affordable, necessary psychotropic medicines, especially in low‐income nations [4]. This article aims to shed lights on the challenges of mental health services in Bhutan and potential ways forward.

## 2 Mental Health Services in Bhutan

After the inception of mental health program in 1997, 8‐bedded Psychiatry Ward was established in 2004. Prior to the establishment of psychiatric ward, clients were treated in general medicine wards [2]. Treating psychiatric clients in general medicine wards could have interfered with patient's safety, confidentiality, and privacy issues making both the patients and the mental health workers uncomfortable. The total bed strength increased to 18 in 2012 and to 20 in 2018 [2]. The Department of Psychiatry, JDW National Referral Hospital is the only psychiatric unit providing specialized mental health services in Bhutan. However, mental health services were gradually expanded to district hospitals and Primary Healthcare Centres (PHCs) through the initiatives of training general doctors, nurses, and other health professionals in mental health [1, 7].

According to WHO‐Assessment Instrument for Mental Health System (WHO‐AIMS 2006), mental health services were instituted to 63 outpatient health facilities in district hospitals and PHCs with a total of 100 beds. Trainings for doctors, nurses, and other health professionals of PHCs were initiated [1, 7]. As of today, all PHCs are supplied with a good range of psychotropic drugs, including antipsychotic, antidepressant, mood stabilizers, antiepileptic, and benzodiazepine drugs, and a few injectable drugs like chlorpromazine, haloperidol, phenytoin, and diazepam [7]. District hospitals and PHCs also render necessary pharmacotherapy and counseling services [1, 2]. Mental health services were expanded to schools by institutionalizing full‐time counselors in schools [8]. Civil Society Organizations (CSOs) such as RENEW (Respect, Educate, Nurture, and Empower Women) and *Nazhoen Lamtoen* also provide counseling services for individuals with mental health issues. Additionally, there are trained counselors in colleges and CSOs who coordinate with the National Mental Health Program to manage mental illness in their settings [1]. There are also dedicated CSOs in the country focused on delivering rehabilitation treatment services, raising awareness, and coordinating psychosocial support [9].

The provision of mental health services in a country requires a collaborative effort of various sectors, including education, health, and CSOs. To ensure improved collaboration and coordination, the need for a lead agency for mental health in a country was felt. Hence, The PEMA, the nodal agency for mental health, was instituted to streamline efforts in planning, implementing, and monitoring mental health policies and services [2, 9]. Under this agency, 60‐beded hospital called The PEMA Center will be built as the apex referral, research, and training center for mental health [2].

The Psychiatry Department consists of multidisciplinary team as reflected in Table 1. It offers both in‐ and out‐patient services including psychiatrist's consultation, psychotherapy, speech and occupational therapy, and various pharmacotherapies [2]. Besides clinical services, it also serves as a consultation and supervising team for the mental health professionals across the country. It also provides training to the resident doctors, medical interns, clinical counselors, and nursing students.

**TABLE 1:** Mental health professionals in Psychiatry Department, Jigme Dorji Wangchuck National Referral Hospital, Bhutan, 2023 [2].

Designation	Numbers in total	Regular/Contract
Psychiatrist	4	2/2
Clinical counselor	4	4/0
Clinical nurse	3	1/2
General Nursing and Midwifery	6	6/0
Occupational therapist	2	2/0
Speech and language pathologist	2	1/1
Health assistant	1	1/0
Peer counselor	4	0/4
Pharmacy technician	1	1/0

## 3 Challenges for Mental Healthcare Services in Bhutan

### 3.1 Geographical Inaccessibility

The health system in Bhutan is structured into three tiers: PHCs at the grassroot, district hospitals at secondary level, and referral hospitals at the tertiary level. There are 179 PHCs, 49 general and district hospitals, and 3 referral hospitals in the country [5]. The Psychiatry Unit at the JDW National Referral Hospital, Thimphu located in the western part of Bhutan renders specialized mental health services. The absence of specialized mental health service in eastern and central regional referral hospital is a major impediment to the efficient delivery of mental health services requiring specialized mental healthcare [1]. However, in one referral and seven district hospitals, there are clinical counselors (psychotherapist) who provide psychotherapy services. As of 2023, there are 16 clinical counselors placed in various districts and organizations [5]. They assess patients with psychiatric issues and provide counseling/psychotherapy. At the primary level, general doctors and health assistant provide mental health services.

The clients from very far‐flung corners of the country requiring psychiatrist consultation and other specialized mental healthcare have to travel for a day or more. They traverse more than 500 km by bus or private car, navigating numerous twists and turns through mountainous roads, whereas a small portion of the population chooses domestic flights [10]. Due to the substantial expenses associated with traveling such great distances, some people consult only when in dire need, leading to limited access to services for certain populations. Additionally for patients residing from places away from the vicinity of clinical counselors, it is difficult for them to avail psychotherapy services. Follow‐up with these groups of patients is a significant challenge.

### 3.2 Human Resource Constraints

The multidisciplinary team in Psychiatry Department, JDW National Referral Hospital lacks psychologist and social workers. It also lacks professionals with subspecialty. Apart from one child and adolescent psychiatrist, there are no psychiatrist, clinical counselors, nurses, and other mental health professionals specialized in various subspecialties. There are no mental health professionals allocated in central and eastern regional referral hospital, district hospital, and in PHCs except the clinical counselors situated in less than half the districts in Bhutan. Moreover, the clinical counselors are all placed in southern and western parts of the country. As of 2023, the number of school counselor stands at 160 serving more than 500 schools [8]. The number of school counselors falls short of the recommended standard, which suggests having one counselor for every 400 students [8]. However, in many schools, there is only single counselor for more than 500 students [8].

### 3.3 Misconception and Stigma Associated With Mental Illness

The conservative ideas about mental illness hinder availing mental health services [1, 11]. In Bhutan, people's attitudes toward mental illness are strongly embedded in their traditional beliefs [11]. Mental illness is believed to be caused by karma, ghosts, and disturbances to the local deities [11]. Widespread myths surrounding mental illness are prevalent and people often associate mental illness with weakness or a lack of willpower, internalizing the triggers and causes, leading to a huge gap in help‐seeking behavior [12]. With those beliefs, a significant fraction of population opts for religious or traditional treatment. Family dynamics, parenting skills among many other causes of psychiatric disorders, are poorly studied in the country. Due to poor mental health literacy and cultural beliefs about mental health, mental illnesses are perceived as “mad” and referred to using derogatory terms like “psycho” or “choelo” meaning mad [11]. They are often stigmatized, marginalized, and neglected creating barriers in seeking professional help.

### 3.4 Inadequate Funding on Mental Health Sector

Inadequate funding in mental health sector is observed worldwide, but the issue is particularly pronounced in low‐ and middle‐income countries [4]. Low‐ and middle‐income countries allocate only between 0.5% and 1.9% of their health budgets to the treatment and prevention of mental illnesses [4]. Bhutan is no exception. Over the last 5 years, the annual budget allocation to mental health program has remained under Nu 3.5 million (US $ ≈ 42,000) a year, which amounts to 1% of the MoHs budget [1]. Moreover, the amount remained constant over the years despite the increase in mental health needs [1]. However, WHO recommends at least 5% of health budget to be allocated in mental health sector [4].

## 4 Way Forward

### 4.1 Strengthen Human Resource

There are no clinical counselors/psychotherapists certified on different subspecialties like dialectical behavioral therapy, eye movement desensitization and reprocessing, and other various therapies. Hence, it is essential to prioritize the training and licensing of counselors in diverse therapeutic approaches. Moreover, it might be opportune to explore the inclusion of psychologists, social workers, and psychiatric nurses to ensure comprehensive patient care. Additionally, there is a pressing need to augment the quantity and scope of mental health professionals, extending beyond the confines of the national hospital's psychiatric unit.

Policies are in place to initiate training of health professionals at PHCs level on mental health but should be materialized sooner [2, 7]. This should include timely clinical supervision and monitoring of services [2]. The school counselors play a vital role in mental health of students [8]. As previously stated, the ratio of counselors to students is below the recommended level [8]. Hence, it is imperative for the relevant authorities to make efforts to enhance both the quality and quantity of school counselors. Conducting timely multistakeholder meetings, supervisions and monitoring of services would further ensure quality services.

### 4.2 Strengthen CMH Services

According to WHO, CMH refers to the services that are provided in the community, outside of a hospital setting [4]. In Bhutan, CMH services were initiated following the formulation of Bhutan's mental health policy [2, 7]. It includes an expansion of mental health services in 63 community‐based psychiatric inpatient units across the country. Different CSOs like RENEW, National Commission for Women, and Children and *Nazhoen Lamtoen* were institutionalized in supports of mental health [7, 9]. MoH initiated public education and awareness campaigns on mental health. In addition, other government agencies as well as CSOs and professional associations have promoted public education and awareness campaigns [7]. Links with other sectors like PHCs/community health, HIV/AIDS, reproductive health, child and adolescent health, substance abuse, child protection, and education were also established [7].

To further strengthen CMH, concerned agencies must monitor, supervise, and follow‐up CMH services in PHCs [2, 7]. The PEMA must monitor and supervise the mental health, suicide, and drug prevention programs at district and PHCs levels [2]. The need of improved collaboration and coordination among various sectors like education, health, and CSOs was observed [9]. MoH and relevant organizations must emphasize on initiating more mental health promotion activities in different institutions and communities. An alternative should also be considered, involving annual screenings for mental health issues nationwide through mental health campaigns, similar to other medical health initiatives.

### 4.3 Increase Mental Health Literacy Among the General Population

Mental health literacy reduces stigma and promotes help‐seeking intentions [1]. Mental health literacy can be improved through advocacy program in schools, different institutes, and through national media platforms [1, 12]. Mental health literacy among students can be also improved through teaching the science behind mental health and its connection to physical health, for example, studying different forms of trauma and how it changes the physical body, such as the brain. Educating children about mental health equips them with the skills to recognize and comprehend their emotions, handle mental health challenges, and prioritize the significance of mental well‐being. This educational approach can effectively diminish the stigma surrounding mental health issues and foster a culture where seeking support is encouraged [12].

### 4.4 Research on Mental Health

There is a dearth of scientific studies on mental health in Bhutan [1]. Scientific study in mental health is imperative in understanding mental health situation of the country and formulate context‐based policies to address the issues. The data further guide to streamline interventions and monitor progress [1, 2]. Employing evidence‐based approaches in planning, decision‐making, and implementation will help to improve the efficiency of resource allocation and enhance the health status and quality of life for individuals facing mental health issues [1]. Therefore, relevant stakeholders and organizations must instigate to collaborate in producing more scientific evidence. It will help to focus and target on identified areas to further enhance the utilization of scarce resources [1].

### 4.5 Funding on Mental Health

On a global scale, mental health funding is notably inadequate [4]. On average, countries worldwide allocate less than 2% of their healthcare budgets to mental health services [4]. In Bhutan, mental health has received only 1% of health's budget for the last two decades [1]. However, WHO recommends at least 5% of total health's budget in mental health [1, 4]. Bhutan has also received mental health fundings from international donors like (UNICEF), WHO, Health Volunteers Overseas, and other donors. An approach to further enhance mental health programs and initiatives is by fostering and capitalizing on partnerships and resources at both domestic and global levels [12]. Mental health funds are needed to implement various mental health programs, human resource capacity building, infrastructure developments, to carry out mental health promotion activities, and exercise various prevention programs. Therefore, budget allocation for mental health must increase to enhance overall mental health services.

### 4.6 Consolidate Strategies to Combat Stigma Associated to Mental Illness

To combat stigmatization against mental illness, concerted endeavor among different sectors is indispensable [1]. Educating the public to inculcate positive perspective about mental illness through various strategies like the use of social media, national television, through arts, and advocacy programs including the people of communities [1, 12]. Enhancing mental health literacy via educational programs that include teaching on mental health and illness can serve as an alternative approach. Integration and strengthening of mental health treatment in other medical departments like emergency, oncology, and physiotherapy may also normalize mental illness treatment.

## 5 Conclusion

Improving accessibility by strengthening mental health services in PHCs, districts, and referral hospitals remains crucial. There is a shortage of mental health professionals nationwide, necessitating efforts to enhance both the quality and quantity of professionals. Adequate funding is essential, as mental health receives only 1% of the health budget, falling short of the WHO's recommendation of 5% health budget. Addressing stigma through enhanced mental health literacy, education, and community involvement is imperative. Mental health services should extend beyond treatment to focus on CMH and collaboration across sectors, while prioritizing evidence‐based practices through increased research efforts.

## Author Contributions

**Kelzang Gyeltshen:** Conceptualization; writing–original draft preparation; writing–reviewing & editing. **Bikram Chhetri:** Conceptualization; writing–reviewing & editing; supervision. **Dawa Gyeltshen:** Conceptualization; writing–reviewing & editing, supervision.

## Ethics Statement

The authors have nothing to report.

## Conflicts of Interest

The authors declare no conflicts of interest.

## Data Availability

The authors have nothing to report.

## References

[1] T. Tsheten , D. Chateau , N. Dorji , et al., “Impact of COVID‐19 on Mental Health in Bhutan: A Way Forward for Action,” The Lancet Regional Health‐Southeast Asia 11 (2023): 100179, 10.1016/j.lansea.2023.100179. 37020787 PMC10008798

[2] C. Dorji , “Addressing Mental Health in Bhutan,” The Druk Journal 9, no. 1 (2023): 4–14, https://drukjournal.bt/wp‐content/uploads/2023/04/Dr‐Chencho‐Dorji.pdf.

[3] A. A. Pollack , H. D. Minh , Nguyen , T. Le , and L. Le , Comprehensive Study on School‐Related Factors Impacting Mental Health and Wellbeing of Adolescent Boys and Girls in Vietnam (New York City: UNICEF, n.d), https://www.unicef.org/vietnam/reports/study‐school‐related‐factors‐impacting‐mental‐health‐and‐well‐being‐adolescents‐viet‐nam.

[4] World Health Organization , Mental Health Atlas 2020 . (Geneva: WHO, 2021), https://apps.who.int/iris/handle/10665/345946.

[5] Ministry of Health , Annual Health Bulletin, Thimphu; PPD, MoH . (Mogadishu: Ministry of Health, 2023), https://www.moh.gov.bt/wp‐content/uploads/Annual‐Health‐Bulleti‐2023.pdf.

[6] K. Tshering and K. Dema , “Psychological Impact During COVID‐19 Pandemic: A Web‐Based Cross‐Sectional Study among Students Studying at College of Science and Technology (CST), Phuentsholing, Bhutan,” PLoS ONE 17, no. 2 (2022): e0263999, 10.1371/journal.pone.0263999. 35176080 PMC8853568

[7] WHO and Ministry of Health , WHO‐AIMS Report on Mental Health System in Bhutan (Thimphu, Bhutan: WHO and Ministry of Health, 2007), https://cdn.who.int/media/docs/default‐source/mental‐health/who‐aims‐country‐reports/bhutan/who/aims/report.pdf?sfvrsn=f082a3c53&download=true.

[8] N. Katel , “Addressing Shortage of Counsellors in Schools,” The Bhutanese. (2023). accessed July 1, 2023, https://thebhutanese.bt/addressing‐shortage‐of‐counsellors‐in‐schools/#:~:text=The%20Ministry%20is%20actively%20working,to%20expand%20the%20intake%20capacity.

[9] The PEMA Secretariate , The Institution of “The PEMA” for Mental Health Care for Bhutan (Thimphu, Bhutan: The PEMA, 2023), http://drukjournal.bt/wp-content/uploads/2023/05/The-Pema-Secretariat.pdf.

[10] Bhutan Transport and Construction Authority , Road Map with distance (Thimpuh, Bhutan: Bhutan Transport and Construction Authority, 2023). accessed April, 2024, https://bcta.gov.bt/bctaweb/load.html?id=95&field_cons=MENU.

[11] D. K. Nirola , J. C. Durham , and K. L. Kraus , “Balancing Traditional Beliefs and Medical Science: Mental Health Care in Bhutan,” Bhutan Health Journal 1, no. 1 (2015): 66–69, 10.47811/bhj.10.

[12] S. Pradhan and U. S. Lhamo , “Global Crisis, National Commitment: Overcoming Challenges to Inclusive Mental Health in Bhutan,” The Druk Journal 9, no. 1 (2023): 71–76, https://drukjournal.bt/wp‐content/uploads/2023/07/Global‐Crisis‐National‐Commitment.pdf.

